# Characterization and assessment of HBV chronically infected patients: Identification of those eligible for treatment in the South West region of Cameroon

**DOI:** 10.1371/journal.pone.0203312

**Published:** 2018-09-05

**Authors:** Kukwah Anthony Tufon, Damian Nota Anong, Henry Dilonga Meriki, Teuwafeu Denis Georges, Mouladje Maurice, Youmbi Sylvain Kouanou, Ayah Flora Bolimo, Nyeke James Tony, Tebit Emmanuel Kwenti, Ndze Henry Wung, Theresa Nkuo-Akenji

**Affiliations:** 1 Department of Microbiology and Parasitology, Faculty of Science, University of Buea, Buea, South West Region, Cameroon; 2 Buea Regional Hospital, Buea, South West Region, Cameroon; 3 Department of Biological science, Faculty of Science, University of Bamenda, Bamenda, North West Region, Cameroon; 4 Department of Public health and Hygiene, Faculty of Health science, University of Buea, Buea, South West Region, Cameroon; 5 BioCollections Worldwide Inc., Miami, FL, United States of America; 6 Department of internal medicine, Faculty of Health science, University of Buea, Buea, South West Region, Cameroon; 7 Department of Medical Laboratory Science, Faculty of Health science, University of Buea, Buea, South West Region, Cameroon; 8 Department of Biochemistry and Molecular Biology, Faculty of Science, University of Buea, Buea, South West Region, Cameroon; Centre de Recherche en Cancerologie de Lyon, FRANCE

## Abstract

**Background:**

The management of patients with chronic hepatitis B infection is quite complex because it requires an in-depth knowledge of the natural history of the disease. This study was aimed at characterizing HBV infected patients in order to determine the phase of the infection and identify the proportion eligible for treatment using 3 different guidelines.

**Methods:**

HBV chronically infected patients (negative for HIV and HCV) were enrolled and the following tests were done for them: ALT, AST, HBV viral load, HBV serologic panel and Full blood count. APRI score was calculated for all patients. These patients were classified into immunotolerant, immune clearance, immune control and immune escape phases of the infection. The WHO and the 2018 AASLD criteria was also used to identify those who need treatment. Patients were clinically examined for signs and symptoms. Questionnaire was administered to all participants to ascertain their treatment status. Statistical analysis was done using SPSS version 21.

**Results:**

A total of 283 participants **(**101 females and 182 males) with a mean age of 31.3±8.5 were enrolled. Fifty-two (18.4%) were eligible for treatment (Immune clearance and immune escape phases) and they recorded a significantly higher mean APRI score (0.71±0.51) as compared to those in the immune control and immune tolerant phase (0.43±0.20). Based on WHO and AASLD criteria, 12(4.2%) and 15 (5.3%) were eligible for treatment respectively and these were all subsets of the 52 cases mentioned above. Six (2.1%) and 29 (10.2%) of those identified under the immune control phase were on tenofovir and traditional medication respectively.

**Conclusion:**

Considering treatment for patients in the immune clearance and immune escape phases of the infection can be a reliable strategy to implement in our setting as this may probably tie with considerations from other treatment guidelines. Fifty-two (18.4%) patients were eligible for treatment and none of them were among the 2.1% of patients put on Tenofovir based treatment. This calls for the need for more trained health experts to periodically assess patients, implement an adequate treatment guideline and place the right patients on treatment in Cameroon.

## Introduction

There are approximately 248 million hepatitis B surface antigen (HBsAg) positive individuals globally [1,2] and about 25% of those infected are liable to liver-related death. The infection happens to be the leading cause of liver cancer worldwide despite the existing measures to curb the morbidity and mortality rate. Chronic HBV infection is known to have different phases which can be identified via the simultaneous use of some virologic, serologic and biochemical markers directly involved in monitoring the progression of the disease. [3]. An understanding of these markers and how they can be exploited in determining the phase of the disease is quite crucial in making relevant treatment and management decisions.

The assessment of patients with chronic hepatitis B (CHB) infection is quite complex because it requires an in-depth knowledge of the natural history of the disease [4]. CHB infection has been classified into different phases which include immunotolerant, immune clearance, immune control and immune escape phase. These phases do not always follow a chronological order and not all chronically infected patients go through all phases. The phases have varying durations and may share a lot in common making it quite complex to distinguish between them in clinical practice [5]. Treatment is usually recommended for patients who are in the immune clearance and immune escape phases of the infection [5–9].

HBV antiviral therapy is meant to prevent, halt or even reverse the progression of liver injury towards cirrhosis, liver decomposition and liver cancer, which are the major causes of HBV related death in older patients with the infection [10,11]. This is achieved by controlling viral replication, either with direct acting antiviral therapy (lamivudine, telbivudine, tenofovir, emtricitabine) or indirectly using interferon (IFN) to stimulate immune control [12,13]. The term “cure” is usually not used in the treatment of CHB infection, given that there is always persistence of covalently closed circular DNA of viral origin in the nucleus of hepatocytes, even in persons with evidence of resolved infection. This poses a lifelong risk of reactivation of the infection [14].

The decision to treat is usually clear in persons who present with life-threatening or advanced liver disease, such as acute liver failure and cirrhosis. Persons with minimal fibrosis and low risk of CHB progression are not usually considered for treatment but such people need to be identified and monitored over time [10].

According to the World Health Organisation (WHO), the following group of people should be treated: (1) Everybody with an Aspartate aminotransferase/platelet ratio (APRI) score >2 (evidence of cirrhosis) irrespective of ALT and HBV DNA levels. (2) Chronically infected adults > 30 years of age with APRI ≤2, persistently abnormal ALT levels and HBV DNA >20 000 IU/mL regardless of HBeAg status [10,15].

The European Association for the Study of the Liver (EASL) and the American Association for the Study of Liver Diseases (AASLD) all have treatment guidelines for CHB infection [16,17] which are generally based on the fact that patients in the immune clearance and immune escape phases of the infection should be considered for treatment. However, the 2018 update of the AASLD guideline [18] recommends treatment for HBeAg (Hepatitis B e antigen) positive patients with ALT value ≥2 times ULN (upper limit normal) and HBV DNA levels >20,000IU/ml as well as for HBeAg negative patients with ALT value ≥2 times ULN and HBV DNA levels ≥ 2,000IU/ml.

The management of HBV infection turns out to be very much of a challenge and this is partly because it may require long term clinical follow up (by a health specialist) in order to identify those who need treatment. Despite being an HBV endemic country, Cameroon does not yet have a national algorithm or guideline for the management and treatment of HBV infected patients. Different international guidelines [10,16–20] have been formulated to address this issue and all of them have as goal to identify and administer treatment to HBV infected patients with significant liver fibrosis, cirrhosis, liver failure or at risk of liver cancer. This study was aimed at characterizing HBV infected patients (based on their viral load, liver aminotransferases, HBeAg status and APRI score) and exploiting some international treatment guidelines in order to identify those that can be more relevant in our setting. This information could be an important tool in the formulation of a national guideline for the management and treatment of HBV infected patients in Cameroon.

## Materials and methods

### Ethical considerations

The National Ethics Committee of Research for Human Health (Number 2016/10/819/CE/CNERSH/SP) and the administrative authority of Buea Regional Hospital approved the study. Each participant submitted a signed consent form before enrolment. The parents or guardians of minors gave their consent before enrolment into the study. For those who could not read nor write, a trained counsellor explained the content of the informed consent and presented the purpose of the research.

### Study design and study area

This was a longitudinal study where HBV chronically infected patients were enrolled and monitored for possible changes 6 months after their enrolment. The study lasted for 5 years (2012–2017) and it was carried out in the Buea Regional Hospital. This is a secondary level reference hospital in Buea, the capital of the South West region of Cameroon. It is a multi-disciplinary hospital that offers cores in surgery, internal medicine, gynaecology/obstetrics, Ophthalmology etc. It has a well-equipped blood bank unit and an ISO 15189 accredited medical laboratory (South African National Accreditation system -SANAS).

### Study population and sampling technique

HBV chronically infected participants (≥ 18 years old) were enrolled from the following groups of people: i) Individuals who came to donate blood at the blood transfusion unit and tested positive for HBsAg. ii) Individuals who came for premarital tests as well as general screening for sexually transmitted diseases (STD) and tested positive for HBsAg. iii) Patients who came for consultation and tested positive for HBsAg following doctor’s request. iv) Admitted patients who tested positive for HBsAg following doctor’s request. v) The snowball sampling technique was also used where already enrolled participants proposed and linked us to other eligible participants from among their acquaintances, friends and family members. vi) Some of the participants were HBsAg positive individuals we identified following a free screening exercise we conducted.

The following people were excluded from the study: (1) Any HBV infected participant co- infected with HIV or HCV. (2) Any pregnant HBV infected female. (3) Any person who did not sign the informed consent form.

### Sample size calculation

The sample size was estimated using the formula for sample size calculation described by Swinscow [21] as follows [22];

$$n=\frac{Z^{2}x\;p\;(1-p)}{e^{2}}$$

Z=1.96

p=PrevalenceofHBVinfectioninCameroon=11.2%

e=errorrate=0.05

$$n=\frac{1.96^{2}x\;0.112\;(1-0.112)}{0.05^{2}}=152.8$$

Thus, we recruited 283 participants to adjust for dropout.

### Clinical evaluation and data collection

An interviewer based standard questionnaire was administered to all participants to find out if they have been on HBV treatment. A medical doctor clinically examined the participants for presence of some signs and symptoms like jaundice, swollen stomach, right upper quadrant abdominal pain, fever, fatigue, loss of appetite, nausea etc.

### Sample collection, processing and laboratory analysis

Venous blood was aseptically collected from all participants by a trained phlebotomist into 2 tubes (5 mls each): one containing Potassium Ethylenediaminetetraacetic acid (K_3_EDTA) and one dry tube with no anticoagulant. The sample from the tube with the anticoagulant was used to perform Full Blood Count (FBC) with the Auto Haematology Analyzer (Mindray model BC-2800, Mindray Bio-Medical Electronics, Nanshan, Shenzhen, P.R. China) following manufacturer’s instructions.

The sample in the tube with the anticoagulant and the sample in the dry tube were then centrifuged at 3000rpm for 5 minutes to obtain plasma and serum respectively. The serum was used for the following tests:

#### HBsAg, HCV and HIV test

Using immuno-chromatographic tests strips (Diaspot for the detection of HBsAg, Acon^®^ Laboratories Inc for HCV antibody detection and Abbot Determine for HIV antibody detection) following manufacturer’s instructions. These tests were done at baseline (enrolment) and after 6 months (follow up visit) for each participant to confirm HBV chronicity (HBsAg positivity for more than 6 months) and negativity for HIV and HCV.

#### Liver aminotransferase measurement

AST [AST-(GOT)-Human reagent] and ALT levels [ALT-(GPT)-Human reagent] were measured by spectrophotometry (Mindray^®^ BA-88 Biochemistry analyser) following the manufacturer’s instructions. Normal value for ALT was <32 U/l for females and <42 U/l for males while normal value for AST was <31 U/l for females and <37 U/l for males. These tests were done at baseline (enrolment) and after 6 months (follow up visit) for each participant to identify possible elevation or changes over time for easy classification into the various CHB phases.

#### HBV serologic profile test

This was done using immunochromatographic panel kit (Blue Cross Bio-Medical Co., Ltd) for the qualitative detection of HBsAg, anti-HBs, HBeAg, anti-HBe and anti-HBc following manufacturer’s instructions.

The plasma obtained after centrifugation was packed and shipped in dried ice (as biological substance category B following international standards) to Biocollections worldwide Miami in USA for the following analysis:

#### ELISA tests for the detection of HBsAg, HIV and HCV antibodies

Shipped plasma specimens were further screened for HBsAg, HCV and HIV using Genetic systems Hepatitis B surface antigen enzyme immuno-assay (Bio-Rad Laboratories, Washington DC, USA), Chiron RIBA HCV 3.0 strip immuno-blot assay (Ortho Diagnostic Systems, New Jersey, USA) and GS Western blot respectively according to manufacturer’s instructions.

#### HBV Viral Load determination

The Abbott Real *Time* HBV automated *m*2000 system was used to extract as well as determine HBV DNA levels following manufacturer’s instruction.

### Calculation and interpretation of APRI score

In order to determine level of fibrosis, APRI score was used [10]. This was calculated by inserting the corresponding AST value, Upper limit normal (ULN) AST value and Platelet count in the following formula [23]:

$$APRI=\frac{AST\;level÷{ULN}^{*}}{Platelet\;count\;(\times 10^{9}/L)}\times 100$$

*ULN is the upper limit normal of AST for the reagent used.

An APRI score >1.5 denoted significant fibrosis (SF), between 0.6 and 1.5 denoted mild fibrosis (MF), between 0.5 and 0.6 denoted progressive fibrosis (PF) and APRI scores <0.5 were considered to have no fibrosis [24,25]. An APRI score of >2 was used to denote cirrhosis [10].

### Classification into the different phases of chronic HBV infection

Chronic HBV infection phases were classified based on Croagh and Lubel [5], EASL [16] and AASLD [18].

*Immune tolerant Phase*: HBeAg-Positive, HBV DNA levels ≥20,000IU/ml, ALT-Persistently normal.

*Immune clearance phase*: HBeAg-Positive or seroconversion transitional stage with HBeAg-negative and HBeAb negative, HBV DNA levels -between 2000 and 20,000 IU/ml, ALT-persistently elevated (Abnormal) or fluctuating (one abnormal and one normal result)

*Immune control phase*: HBeAg-negative, HBeAb-Positive, HBV DNA levels <2000 IU/ml, ALT-persistently normal.

*Immune escape phase*: HBeAg-negative, HBeAb-Positive, HBV DNA levels ≥2000 IU/ml, ALT-persistently elevated (Abnormal).

### Identification of CHB patients who need treatment based on WHO guideline

HBV infected participants were divided into 2 groups based on their APRI score value (<2.0 and ≥ 2.0). Those who had APRI score value ≥ 2.0 were considered fit for treatment. Those with APRI<2.0 were further divided into two groups based on their ages (≤30 years and >30 years of age). Those who were >30 years of age and who had abnormal ALT (higher than upper limit normal) and HBV DNA levels >20,000 IU/ml were considered fit for treatment [10].

### Identification of CHB patients who need treatment based on AASLD guideline

HBV infected participants were divided into 2 groups based on their HBeAg status. The participants with ALT values ≥ 2 times ULN (Upper limit normal of ALT for healthy adults) were selected from amongst the HBeAg positive and HBeAg negative participants. HBeAg positive participants with ALT values ≥ 2 times ULN and HBV DNA levels ≥20,000IU/ml were considered eligible for treatment. HBeAg negative participants with ALT values ≥ 2 times ULN and HBV DNA levels ≥2000 IU/ml were also considered eligible for treatment [18].

### Statistical analysis

Data was analyzed using SPSS version 21.0 (Statistical Package for the Social Sciences, Chicago, Illinois) and presented as number of cases, percentages and mean ± standard deviation (SD). Categorical comparisons were done using Chi-square test and a two-sided p value < 0.05 was considered significant for all analyses.

## Results

**Fig 1:**
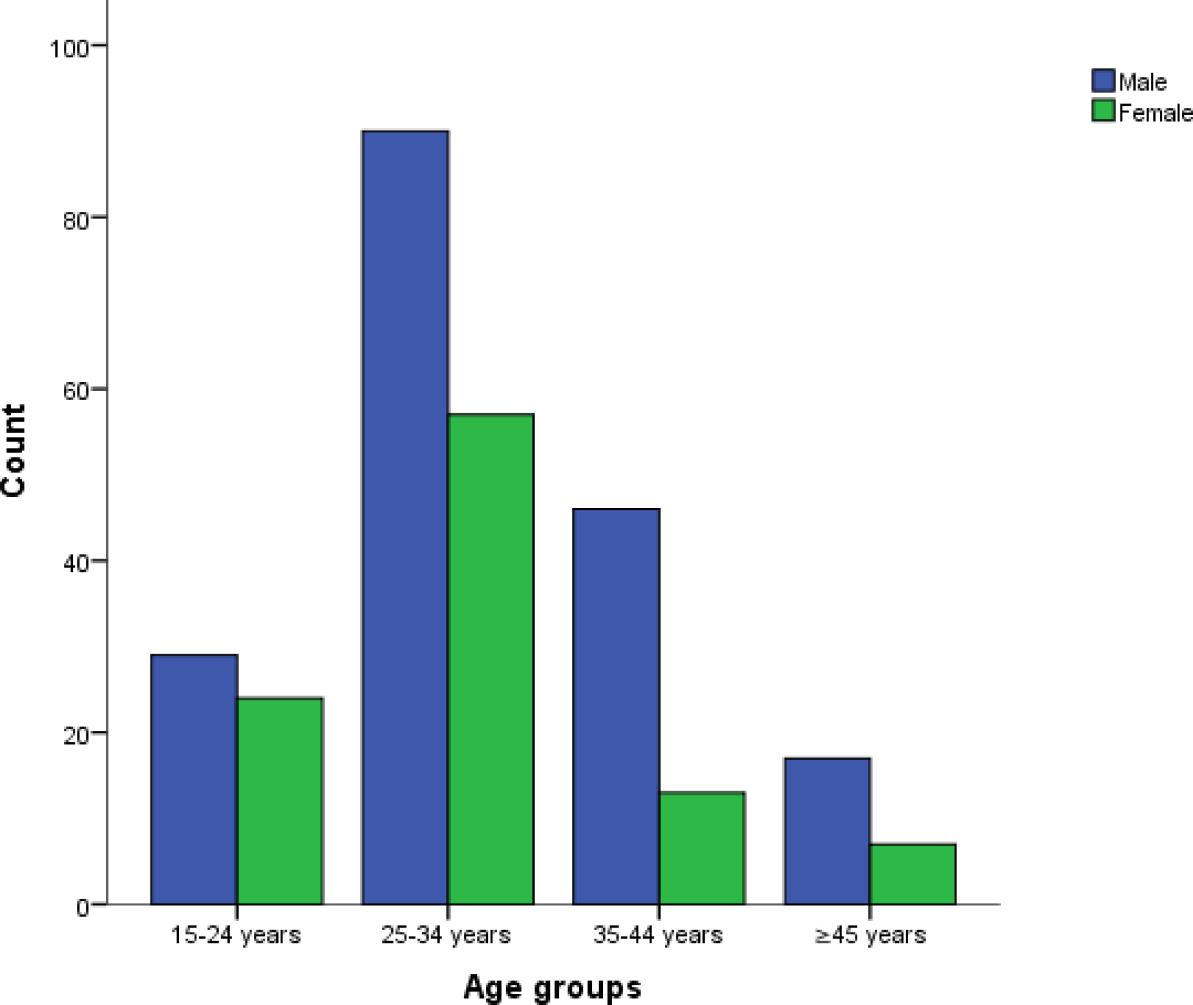
Age and gender distribution.

A total of 292 HBV infected participants were enrolled in this study although we subsequently excluded 6 for being HIV positive and 3 for being HCV positive. No result discrepancy was recorded between the immunochromatographic strip tests and the confirmatory ELISA tests for the detection of HBsAg and antibodies against HIV and HCV. The remaining 283 had a mean age of 31.3±8.5 with ages ranging from 18 to 62. This population was made up of 101 females (35.7%) and 182 males (64.3%). Females recorded a mean age of 29.3±7.8 years while males had 32.4±8.6 years. Age and gender distribution is presented in Fig 1. Table 1 summarises the results obtained from different parameters tested.

**Table 1:** Summary of tests results.

Test parameters		Females, n = 101	Males, n = 182	General, n = 283
**HB (g/dl)**	Mean ± SD	12.7±1.9	15.1±1.7	14.2±2.1
	Median	12.9	15.2	14.5
	Minimum	6.7	7.7	6.7
	Maximum	17.9	19.5	19.5
**WBC (10^3^/μl)**	Mean ± SD	5.2±1.4	4.9±1.3	5.0±1.4
	Median	4.9	4.7	4.7
	Minimum	2.7	3.2	2.7
	Maximum	9.3	12.0	12.0
**Platelet (10^3^/μl)**	Mean ± SD	251.2±67.5	200.1±50.5	218.8±62.3
	Median	244.5	202.0	211
	Minimum	125	104	104
	Maximum	483	344	483
**ALT (U/L)**	Mean ± SD	24.1±15.1	29.3±19.6	27.4±18.3
	Median	21.0	24.0	22.5
	Minimum	6.0	6.0	6.0
	Maximum	89.0	172.0	172.0
**AST (U/L)**	Mean ± SD	28.2±22.6	31.1±22.2	30.1±22.4
	Median	25.0	29.0	27.0
	Minimum	6.0	7.0	6.0
	Maximum	214.0	254.0	254.0
**HBV Viral load (Log IU)**	Mean ± SD	3.09±1.64	2.87± 1.33	2.95±1.45
	Median	2.85	2.62	2.70
	Minimum	1.0	1.0	1.0
	Maximum	8.31	8.44	8.44

Patients with elevated ALT and elevated AST recorded a significantly higher mean viral load (log IU) as compared to patients with normal ALT and normal AST respectively as seen in Table 2.

**Table 2:** HBV viral load (log IU) across gender, age and liver aminotransferase.

Patient demography		n	Mean HBV viral load (Log IU) comparison			
			Mean ± SD	Standard error	95% CI*	P-value
**Gender**	Male	182	2.87 ± 1.33	0.180	-0.13 to 0.57	0.222
	Female	101	3.09 ± 1.64			
**Age (years)**	≤30	157	2.95 ± 1.44	0.174	-0.34 to 0.34	1.000
	>30	126	2.95 ± 1.48			
**Liver aminotransferases**						
**AST**	Normal	216	2.83 ±1.36	0.200	0.11 to 0.89	**0.013**
	Elevated	67	3.33 ± 1.65			
**ALT**	Normal	233	2.75 ±1.23	0.216	0.75 to 1.60	**<0.001**
	Elevated	50	3.92 ± 1.96			

*Confidence interval

HBV viral load <2000 IU/ml was recorded in 197 participants. Forty-seven participants had HBV viral load between 2000 and 20,000 IU/ml while 39 participants had values >20,000IU/ml.

There was a significantly higher proportion of patients with elevated liver aminotransferases (ALT and AST) having viral loads >20,000IU/ml as compared to the proportion of patients with normal liver aminotransferases having viral loads >20,000IU/ml (Table 3)

**Table 3:** Grouped HBV viral loads across gender, age and liver aminotransferase.

Patient demography		HBV DNA levels (IU/ml)							
		Comparison between cases with <2000IU/ml and 2000–20,000IU/ml				Comparison between cases with <2000IU/ml and 20,000IU/ml			
		n	<2000	2000–20,000	P-value	n	<2000	>20,000	P-value
**Gender** **n (%)**	Male	160	128 (80.0)	32 (20.0)	0.687	150	128 (85.3)	22 (14.7)	0.310
	Female	84	69 (82.1)	15 (17.9)		86	69 (80.2)	17 (19.8)	
**Age** **n (%)**	≤30 years	137	113 (82.5)	24 (17.5)	0.434	133	113 (85.0)	20 (15.0)	0.484
	>30 years	107	84(78.5)	23 (21.5)		103	84 (81.6)	19 (18.4)	
**Liver aminotransferases**									
**AST** **n (%)**	Normal	192	157 (81.8)	35 (18.2)	0.432	181	157 (86.7)	24 (13.3)	**0.014**
	Elevated	52	40 (76.9)	12 (23.9)		55	40 (72.7)	15 (27.3)	
**ALT** **n (%)**	Normal	213	176 (82.6)	37 (17.4)	**0.050**	196	176 (89.8)	20 (10.2)	**<0.001**
	Elevated	31	21 (67.7)	10 (32.3)		40	21 (52.5)	19 (47.5)	

**Fig 2:**
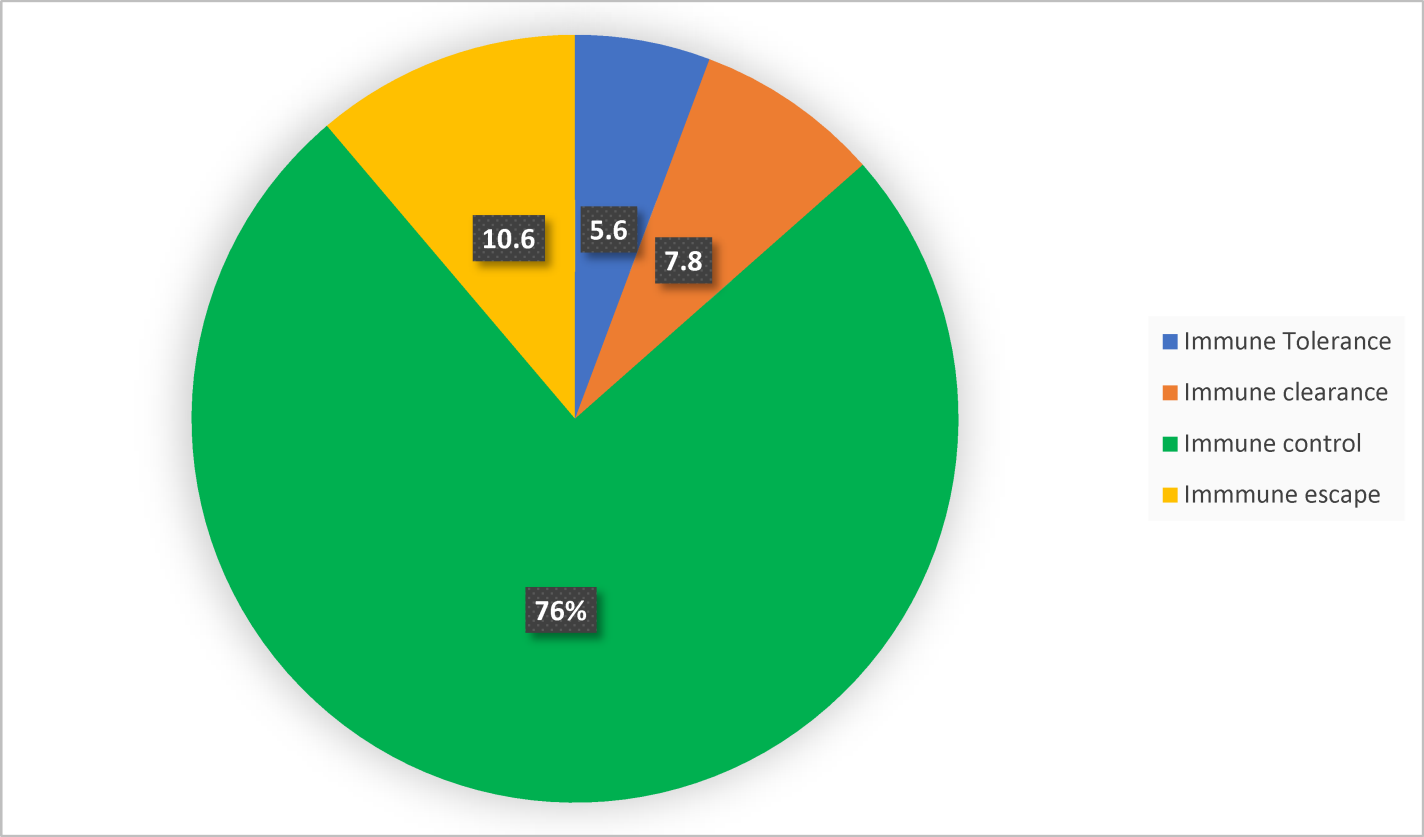
Classification into the different chronic hepatitis B phases.

Following the classification into the four different phases of chronic HBV infection, 215 (76%) of the participants were found to be at the immune control phase of the infection (Fig 2). Only 52 (18.4%) of the participants fell under the category of individuals who should be considered for treatment (immune clearance and immune escape phases).

**Fig 3:**
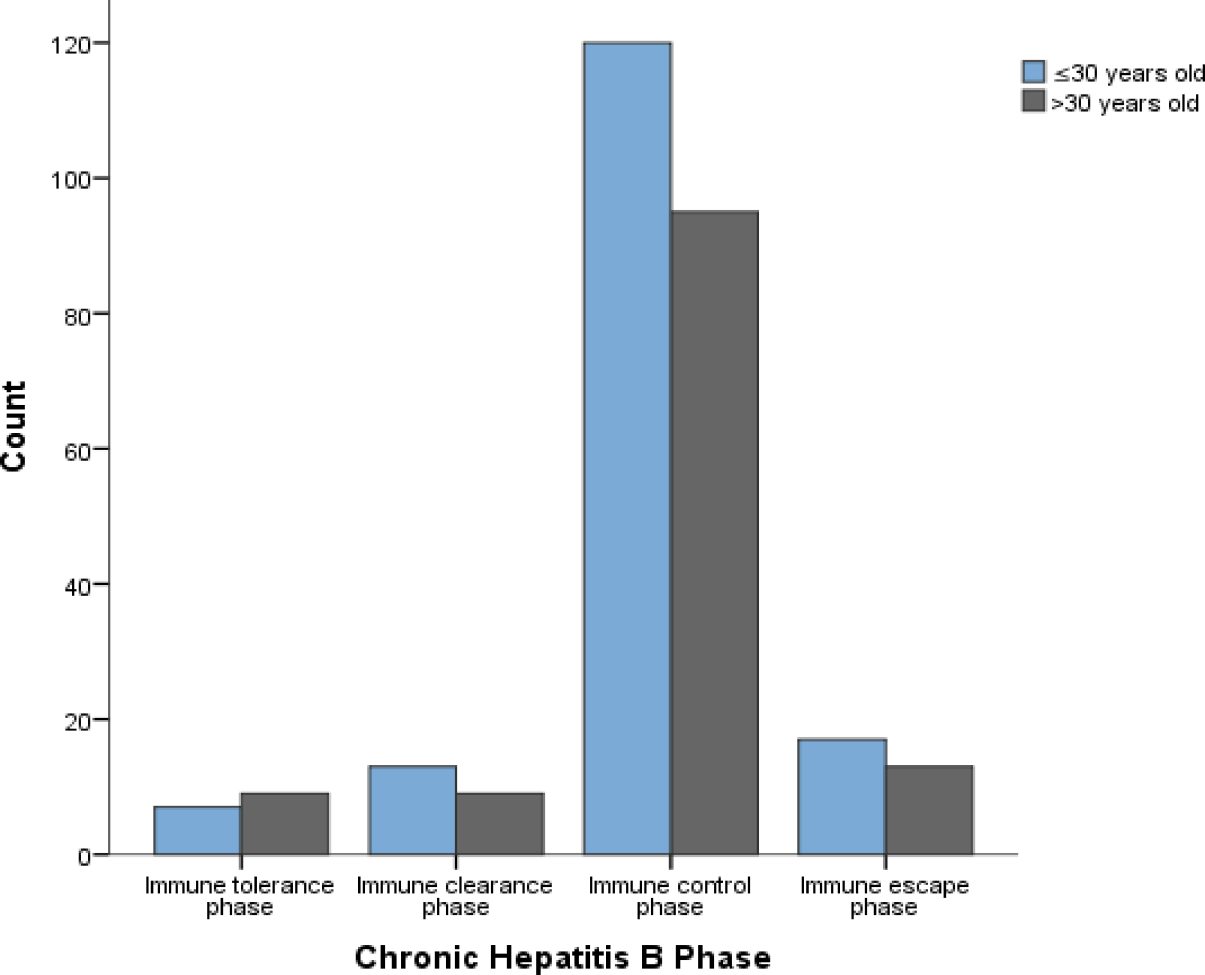
Grouped age distribution across the different CHB phases.

Fig 3 shows age distribution (≤30 years of age and >30 years of age) across the different CHB phases.

Seventeen (6%) out of the 283 HBV infected participants showed signs and symptoms of disease. Majority (8) of those presenting clinical symptoms were at the immune clearance phase. The immune control and immune escape phases each had 4 cases with signs and symptoms while immune tolerance phase had 1 case. The most recurrent clinical symptoms recorded were jaundice, swollen stomach and fatigue.

At the time of enrolment, 85.2% of the participants were not on any form of treatment. Majority of those on treatment were on traditional medication (Table 4). Six (2.1%) of the patients were on tenofovir treatment and all of them happened to be at the immune control phase of the infection. Out of the 29 patients on traditional remedies, 26 (89.7%) were in the immune control phase, 1(3.4%) in the immune tolerant phase and 2 (6.9%) in the immune escape phase.

**Table 4:** HBV infected participants on treatment.

Treatment	Number of participants	Percentage
Tenofovir (Hepazol)	6	2.1
Traditional remedies	29	10.2
Immune boosters	7	2.5
None	241	85.2
***Total***	283	100

**Fig 4:**
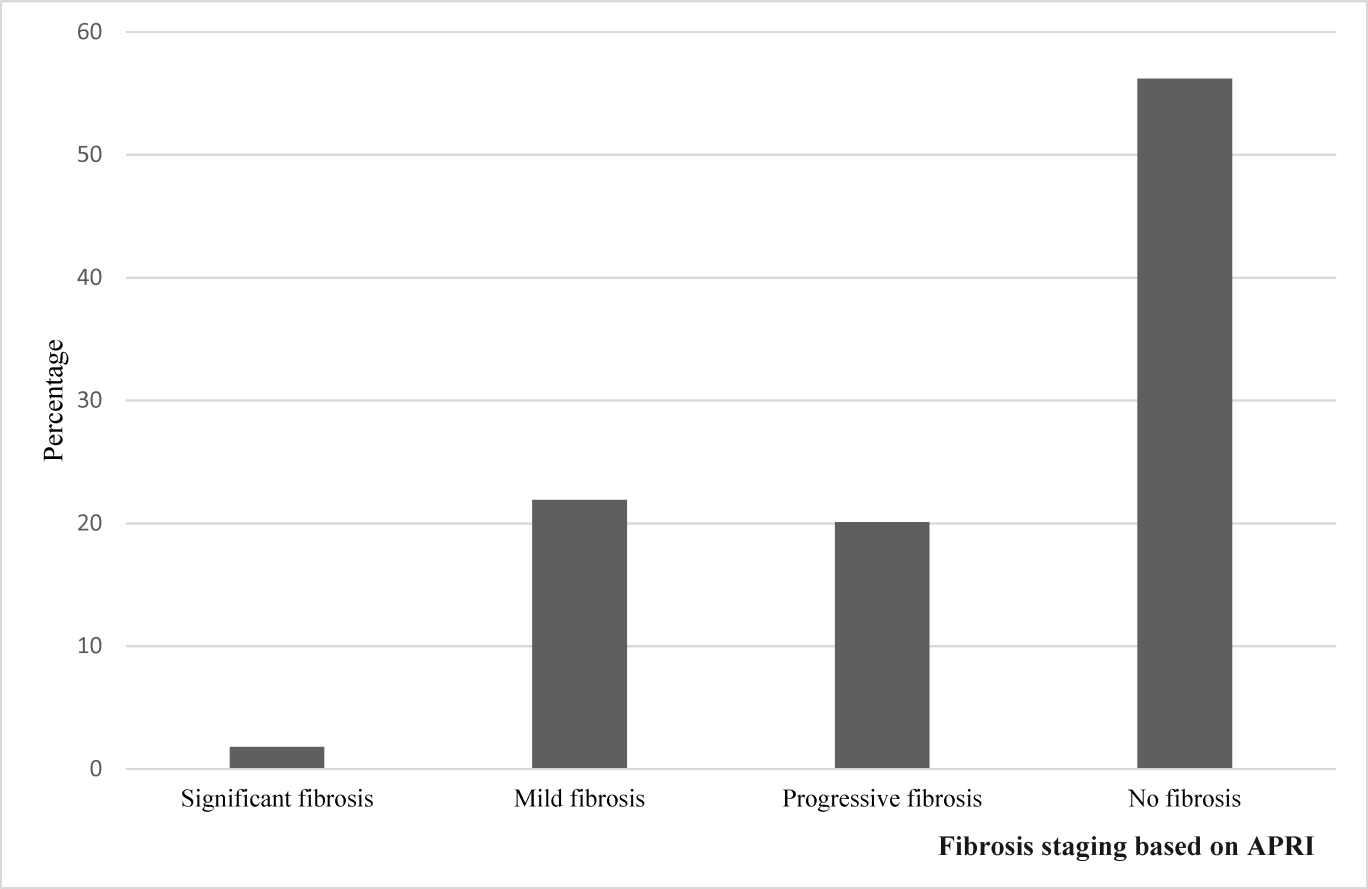
Degree of fibrosis based on APRI score.

A mean APRI score of 0.50±0.32 was recorded for all the participants with scores ranging from 0.12 to as high as 3.10. Majority (56.2%) of the participants showed no evidence of fibrosis based on their APRI score (Fig 4).

The participants who were eligible for treatment based on CHB phase classification recorded a significantly higher mean APRI score when compared to those who were not eligible for treatment (Table 5)

**Table 5:** Chronic hepatitis B phase and APRI score.

CHB Phase of infection		APRI score						
		Stage of Fibrosis				Mean score ±SD		
		Fibrosis			No Fibrosis	Per phase	Grouped*	P-Value
		SF	MF	PF				
Treatment not recommended	**Immune tolerance (16), n (%)**	0	1 (6.2)	6 (37.5)	9 (56.3)	0.33±0.13	0.43±0.20	**<0.001**
	**Immune control (215), n (%)**	0	21 (9.8)	26(12.1)	168 (78.1)	0.44±0.20		
Treatment recommended	**Immune clearance (22), n (%)**	0	12(54.5)	6 (27.3)	4 (18.2)	0.66±0.27	0.71±0.51	
	**Immune escape (30), n (%)**	3 (10)	12 (40)	10(33.3)	5 (16.7)	0.76±0.63		

SF: Significant fibrosis, MF: Mild fibrosis, PF: Progressive fibrosis

*Grouped based on treatment necessity (Immune tolerance +immune control and immune clearance +immune escape)

**Fig 5:**
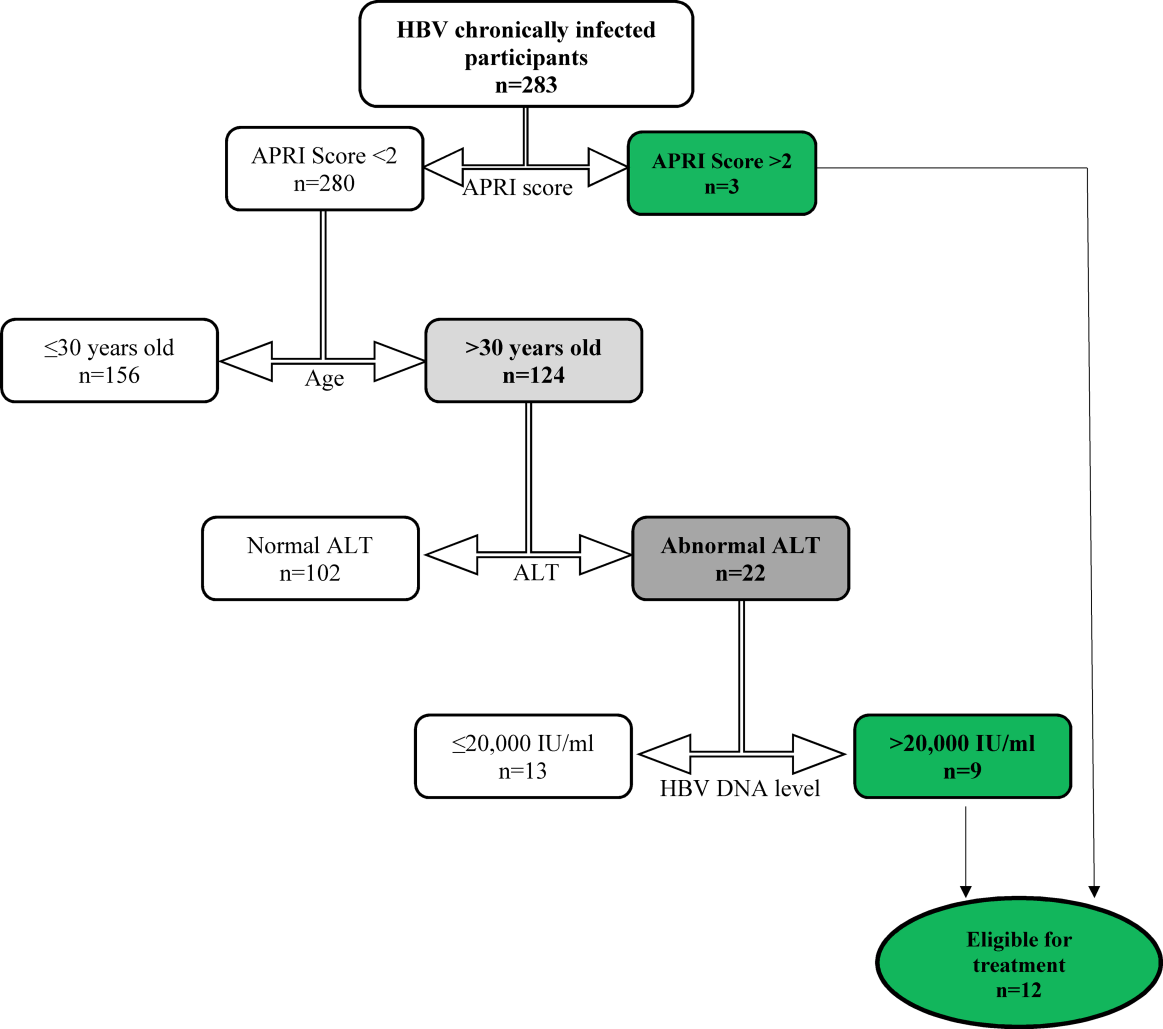
Participants eligible for treatment based on WHO guidelines.

Only 3 cases had APRI score value >2 (possible cirrhosis). Based on WHO guidelines, 12 (4.2%) out of the 283 participants were eligible for treatment (Fig 5).

**Fig 6:**
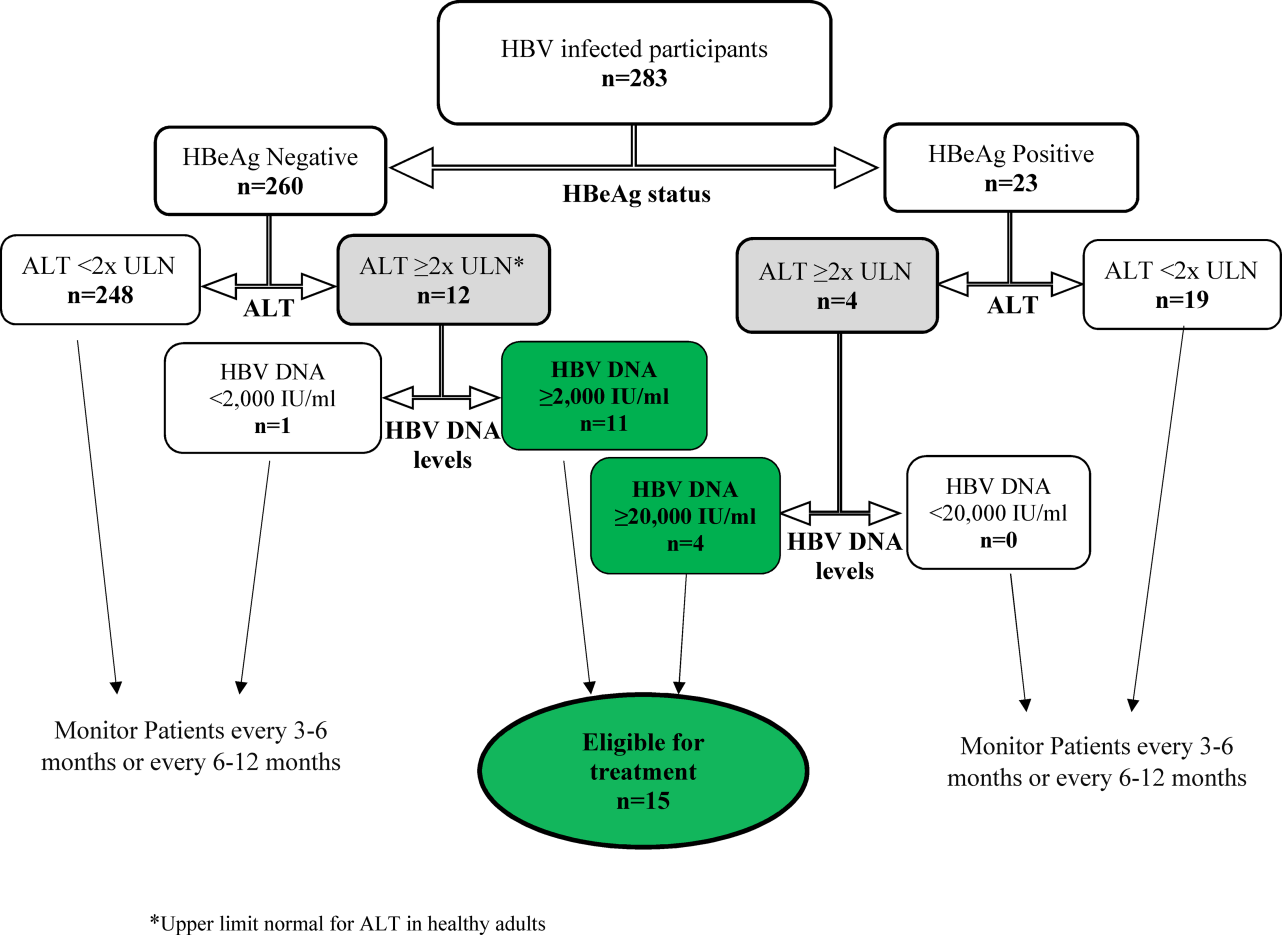
Participants eligible for treatment based on the 2018 AASLD guideline.

Based on the 2018 AASLD guideline, 15 (5.3%) of the 283 participants were eligible for treatment (Fig 6).

**Fig 7:**
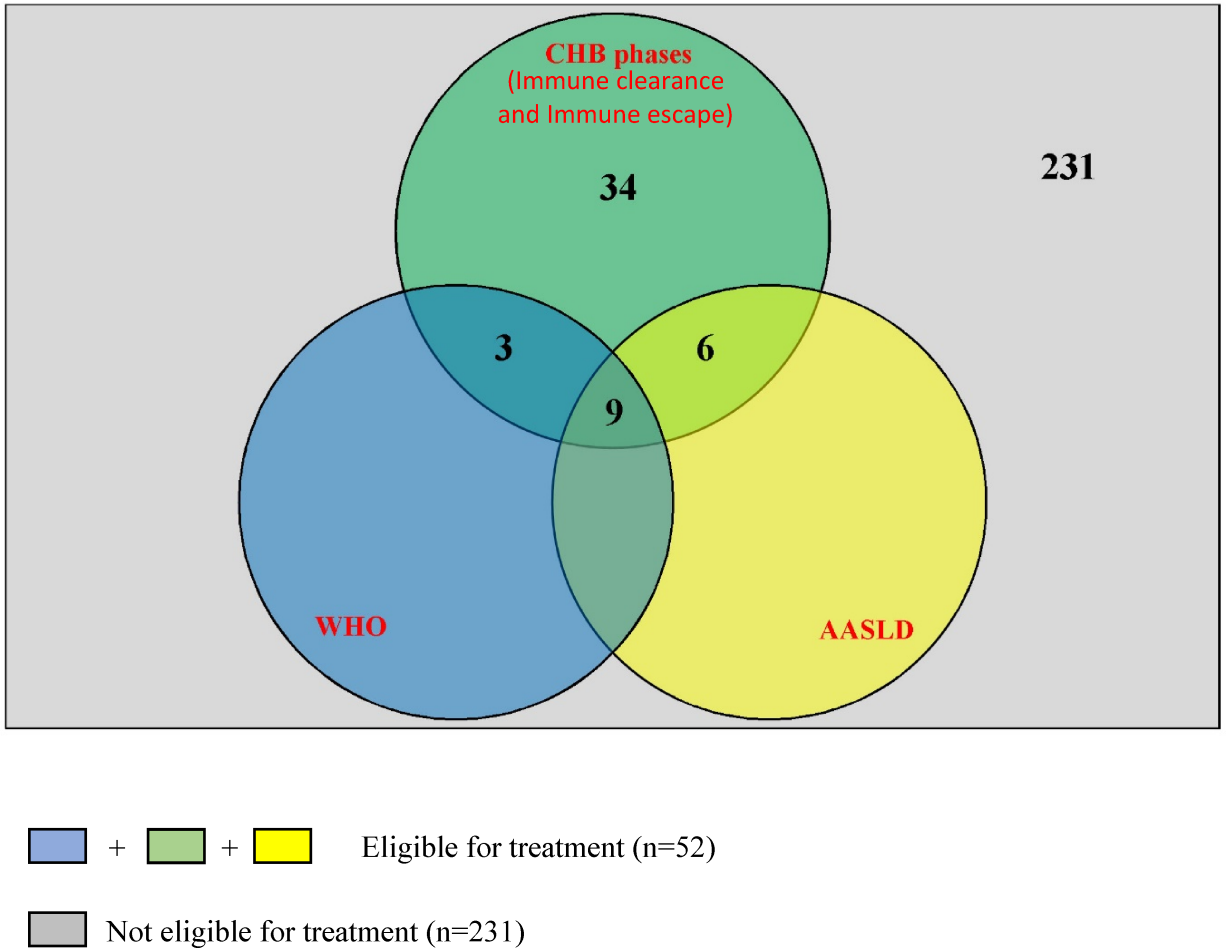
Relationship between the different guidelines used to determine treatment eligibility among the 283 participants.

All the participants eligible for treatment with the 2018 AASLD and WHO guidelines were also part of the 52 cases eligible for treatment based on chronic HBV phases (immune clearance and immune escape phases) as seen in Fig 7.

## Discussion

HBV infection is known to affect millions of people worldwide cutting across all age groups and gender. About 2/3 of the study population happened to be males as they dominated in all the different age groups in this study. Other studies have as well shown that there are more HBV infected males than females [26–28]. Some studies have also shown that males are even more prone to liver disease complications when compared to females and this has nothing to do with behavioural or environmental risk factors [29]. Some hormones like estrogen produced mainly during the reproductive ages of females have been suggested to play a protective role in females when it comes to contracting the disease or developing complications following contraction of the disease [26,30]. In addition to this, some researchers in China noticed the presence of some abnormal liver apolipoprotein found only in males which may be acting as a route to facilitate the contraction or the development of complications once the disease has been contracted [31].

The classification of CHB patients into 4 different phases (immune tolerance, immune clearance, immune control and immune escape phases) can be used as a guide in determining treatment necessity [17]. The challenge for the clinician is to determine the phase of the infection and anticipate the natural history for an individual patient, so that therapy can be directed to those likely to benefit. Out of the 283 participants in this study, 215 (76%) of them fell under the immune control phase also referred to as the inactive carrier phase. Although this huge proportion is similar to what some studies have shown [17,32,33], others had very low proportion of patients classified under the immune control phase [34]. In addition to the fact that the study sites/populations differ, some of these discrepancies may come about because there is still a controversy regarding the HBV DNA cut off point when classifying patients under the immune control or inactive carrier phase of the disease. Some researchers consider HBV DNA levels <2000IU/ml while others consider <20,000IU/ml. We considered HBV DNA levels <2000IU/ml and recorded a percentage as high as 76%. The high percentage of patients in the immune control phase also tells us that a good number of HBV infected people in our study area are HBeAg negative, have very low viral loads and should not be considered for treatment since they probably have minimal or no liver injury. Interestingly, the results we had from APRI actually tied with this concept as majority (56.2%) of our participants had no evidence of fibrosis based on APRI.

Our study showed that participants who were in the immune clearance and immune escape phases had a significantly higher APRI score (most likely to have fibrosis) than those in the other phases. This further explains why treatment should be considered for people who fall in the immune clearance and immune escape phases as they are more at risk of developing cirrhosis and HCC [5,9,35]. Our study identified 22 and 30 participants in the immune clearance and immune escape phases respectively. This made a total of 52 (18.4%) patients who should be considered for treatment. Unfortunately, none of these patients in question were on any form of treatment at their time of enrolment into this study. Instead, all the participants who happened to be on some form of treatment prescribed by a medical doctor (Antiviral Nucleotide analogs or Interferon) during this study were all in the immune control phase of the infection.

HBV infection turns out to be asymptomatic in most HBV infected patients as seen in our study and other studies [36–38]. Our study identified only 17(6%) symptomatic infected cases with majority of them being in the immune clearance phase (necessitating treatment) implying that presence of signs and symptoms may as well play a role in determining whether a patient needs treatment or not. Unfortunately, the asymptomatic nature of the disease that can persist for several years makes it very difficult to identify people who have the disease (or people who are at risk of having the disease) talk less of people who need treatment. In fact, the asymptomatic nature of the disease gives us enough reason to believe that the known prevalence of the disease in Cameroon may actually be an underestimation.

Cameroon’s minister of Public health signed a communique in July 2016 reducing the prices of HBV and HCV treatment and stated that the treatment centres as of that time would all be based in Douala and Yaoundé. This happens to be a good move because HBV infection is more common among people of low social status characterized with poverty and unemployment [39]. However, the limited treatment centres here is a major challenge. Medically trained specialist (gastroenterologist and hepatologist) in the field of liver disease are quite few in Cameroon and majority of them are based in Douala and Yaoundé making it difficult for everyone across the national territory to have access to them in time or whenever the need arises. As a result, Most HBV infected patients have access only to healthcare workers who are not specialist in the field and who may end up taking irrelevant decisions like prescribing antiviral therapy to patients who are inactive carriers (immune control phase).

Some researchers believe that the descriptions given to the 4 identified phases (immune tolerant, Immune clearance, immune control and immune escape) are not fully supported by immunological data [40]. The phases have been shown to have variable duration and are not necessarily sequential. Moreover, they do not always relate directly to criteria and indications for antiviral therapy [10]. For these reasons, WHO modified and came up with new treatment eligibility criteria in 2015 and based on these criteria, only 12 (4.2%) of our participants were eligible for treatment (Fig 5). A study carried out in The Gambia had a similar proportion (4.4%) of HBV infected patients eligible for treatment although they used a different criteria to identify the cases [41]. The 12 cases eligible for treatment in our study based on WHO guideline also happened to be part of the 52 cases earlier identified as people eligible for treatment based on the CHB phases. The difference in number between the two criteria is quite significant and judging from the WHO guideline, very few people would at any given point in time, be eligible for treatment. This could be because WHO guideline considers cirrhosis only when APRI score >2 and very few people had scores higher than 2 in our study. A study by Li *et al* in 2017 [3] showed that APRI >2 cannot be used to represent cirrhosis especially in HBeAg negative patients who have ALT values ≤2 ULN (upper limit normal) as this greatly underestimates the number of cases with cirrhosis. Majority of the HBsAg positive participants in our study happened to be HBeAg negative with ALT values ≤2 ULN. Based on this, using an APRI score >2 happens not to be an appropriate criterion to depict cirrhosis or determine treatment necessity in our study population.

All the participants eligible for treatment based on the 2018 AASLD and the WHO guidelines were either in the immune escape or the immune clearance phase of the infection. This implies that according to our study, considering treatment for HBV infected patients in the immune escape and immune clearance phases of the infection can be a useful and relevant decision since other treatment guidelines would most likely recommend treatment for these patients as well. However, it may not necessarily be the case for all patients because the decision to treat some patients may require the expertise of the health specialist and some complex considerations (age of the patient, family history of hepatocellular carcinoma, risk of transmission, extrahepatic manifestations, etc.) that can only be assessed when patients are followed up individually especially for patients who do not really fall into any of the phases or patients who cannot be clearly categorized based on their results [16]. Unfortunately, we did not take into account some of these conditions which if met, may require that we consider treatment even for a patient in the immune control phase (inactive carrier) [16]. Another limitation for our study could be the fact that we had just one HBV viral load results (at baseline) for all the patients. A follow-up for both HBV viral load and liver aminotransferases over a period of 6 months or more is usually considered when classifying chronic HBV infected patients into the different phases [16,17].

In conclusion, only 52 (18.4%) patients were eligible for treatment and none of them were among the 2.1% of patients put on tenofovir based treatment. Considering treatment for patients in the immune clearance and immune escape phases of the infection can be a reliable strategy to implement in our setting as this may probably tie with the considerations from other treatment guidelines (WHO and AASLD). Identifying CHB infected patients who need treatment is quite a challenge in Cameroon bearing in mind that this exercise requires a series of tests which must be done periodically and interpreted by a specialist simultaneously before a conclusion can be arrived at. Some of these tests are quite expensive and not very much common in our setting. Majority of HBV infected patients in our study population do not need antiviral therapy but need to be engaged in a life time follow-up commitment with a trained health specialist given that hepatitis B is unstable and can change its phase or inactivate at any time. Unfortunately, the recommended health specialist for this purpose are quite few in Cameroon. The health sector needs to train more specialist in liver disease management and distribute them over the national territory so that CHB infected patients can easily have access to them. This would increase the chances of identifying (in time) those who need treatment and those who need monitoring. This would also increase the chances of providing relevant counselling and advice to all CHB infected patients and thus improve on the control and management of the disease.

## Supporting information

S1 File Questionnaire. (PDF)

S2 File Raw data files. (XLSX)

## Abbreviations

HBV: Hepatitis B virus
Anti-HBs: Hepatitis B surface antibody
HBsAg: Hepatitis B surface antigen
HBcAb (anti-HBc): Hepatitis B core antibody
HBeAg: Hepatitis B e antigen
WHO: World Health Organisation
AASLD: American Association for the Study of Liver Diseases
EASL: European Association for the Study of the Liver
HIV: Human Immunodeficiency virus
HCV: Hepatitis C virus
SPSS: Statistical Package for Social Sciences
NECRHH: National Ethics Committee of Research for Human Health
IFN: interferon
ULN: upper limit normal
AST: Aspartate aminotransferase
ALT: Alanine aminotransferase
CHB: Chronic HBV infection
APRI: Aspartate-platelet ratio index
DNA: Deoxyribonucleic acid
